# Supplementary material for: Impact of supervisory behavior on sustainable employee performance: Mediation of conflict management strategies using PLS-SEM
Source: PLoS One. 2020 Sep 2;15(9):e0236650. doi: 10.1371/journal.pone.0236650 (PMC7467322; doi:10.1371/journal.pone.0236650)
Supplement: S1 Data — (DOC) [file pone.0236650.s001.doc]

**Questionnaire**

**Supervisory Behavior and Employee Performance**

This research is to study the relationships among supervisory behavior conflict management strategies and sustainable employee performance. This is purely an academic exercise, and there are no right or wrong answers. Confidentiality is highly assured, so kindly spend some of your precious time to respond to the following questions.

**Section 1 - Consent**

I hereby fill this survey and give my consent to use the information for this study (kept confidential)

|  | Yes |  | NO |
| --- | --- | --- | --- |

**Section 2 – Personal Credentials**

(Your personal credentials will be kept fully confidential and used for this research only)

Gender

|  | Male |  | Female |
| --- | --- | --- | --- |

***Industry***

|  | Manufacturing |  | High-Tech |  | Construction |  | Services |
| --- | --- | --- | --- | --- | --- | --- | --- |

***Experience***

|  | 1-5 years |  | 6-10 years |  | 11-15 years |  | More than 15 years |
| --- | --- | --- | --- | --- | --- | --- | --- |

Age

|  | 20-30 years |  | 31-40 years |  | 41-50 years |  | Above 50 years |
| --- | --- | --- | --- | --- | --- | --- | --- |

**Section 3 - Supervisory Behavior**

How do you rate your supervisor/manager/leader's behavior?

Please note for following questions:

(1= Strongly Agree, 2= Agree, 3= Neutral, 4= Disagree, 5= Strongly Disagree)

| Descriptions | 1 | 2 | 3 | 4 | 5 |
| --- | --- | --- | --- | --- | --- |
| My supervisor/manager is friendly and easy to approach |  |  |  |  |  |
| My supervisor/manager is eager to recognize and reward good performance |  |  |  |  |  |
| My supervisor/manager is willing to listen to my problems |  |  |  |  |  |
| My supervisor/manager treats me with respect |  |  |  |  |  |
| My supervisor/manager is does not control everything, he has confidence in my judgement |  |  |  |  |  |
| My supervisor/manager sets specific goals for me to accomplish |  |  |  |  |  |
| My supervisor/manager emphasizes high standards of performance |  |  |  |  |  |
| My supervisor/manager stresses high standards of performance for group/unit |  |  |  |  |  |

**Section 4 - Conflict Management Strategies**

In case of any conflict at my workplace how do you react?

| Descriptions | 1 | 2 | 3 | 4 | 5 |
| --- | --- | --- | --- | --- | --- |
| ***Competing Strategy*** | | | | | |
| I argue my case with my coworkers to show the merits of my position. |  |  |  |  |  |
| I am firm in pursuing my side of the issue. |  |  |  |  |  |
| I hold on to my solution to a problem |  |  |  |  |  |
| ***Compromising Strategy*** | | | | | |
| I negotiate with my coworkers so that a compromise can be reached |  |  |  |  |  |
| I accommodate the wishes of my co-worker |  |  |  |  |  |
| I propose a middle ground for breaking dead lock. |  |  |  |  |  |
| ***Collaborating Strategy*** | | | | | |
| I try to investigate an issue with my co-workers to find a solution acceptable to us. |  |  |  |  |  |
| I use give and take so that a compromise can be made. |  |  |  |  |  |
| I try to bring all our concerns out in the open so that the issues can be resolved in the best possible way. |  |  |  |  |  |
| ***Avoiding Strategy*** | | | | | |
| I attempt to avoid discussing about my conflicts and try to keep my conflict with my co-workers to myself |  |  |  |  |  |
| I exchange accurate information with my co-workers to solve a problem together |  |  |  |  |  |
| I try to keep my disagreements with my co-workers to myself in order to avoid hard feelings. |  |  |  |  |  |
| ***Accommodating Strategy*** | | | | | |
| I satisfy the expectations of my co-workers. |  |  |  |  |  |
| I avoid open discussion of my differences with my coworkers. |  |  |  |  |  |
| I go along with the suggestions of my co- workers |  |  |  |  |  |

**Section 5 - Sustainable Employee Performance**

To improve your performance at work what do you think about following questions:

| Descriptions | 1 | 2 | 3 | 4 | 5 |
| --- | --- | --- | --- | --- | --- |
| ***Contextual performance*** | | | | | |
| I taking initiatives to maintain my performance at work |  |  |  |  |  |
| I have an attitude of accepting and learning from feedback |  |  |  |  |  |
| I usually cooperate with others |  |  |  |  |  |
| I try to communicate effectively (e.g., adequately expressing ideas and intentions) |  |  |  |  |  |
| ***Adaptive performance*** | | | | | |
| I usually showing resiliency (coping with stress, difficult situations and adversities) |  |  |  |  |  |
| I prefer coming up with creative solutions to novel, difficult problems |  |  |  |  |  |
| I try to keep my job knowledge up-to-date |  |  |  |  |  |
