# Supplementary material for: Impact of supervisory behavior on sustainable employee performance: Mediation of conflict management strategies using PLS-SEM
Source: PLoS One. 2020 Sep 2;15(9):e0236650. doi: 10.1371/journal.pone.0236650 (PMC7467322; doi:10.1371/journal.pone.0236650)
Supplement: S1 Table — (DOCX) [file pone.0236650.s003.docx]

| **Annexure 1:** *Proposed Hypothesis* | | |
| --- | --- | --- |
| **Hypothesis** | **Abbreviations** | **Statements** |
| Hypothesis 1 | (H1) | SB positively and significantly impacts SEP. |
| Hypothesis 2 | (H2) | SB has a positive and significant link with CMS. |
| Hypothesis 3 | (H3) | CMS positively and significantly affect SEP. |
| Hypothesis 4 | (H4) | CMS positively and significantly mediates the relationship between SB and SEP. |
| Hypothesis 5a | (H5a) | Competing CMS positively and significantly mediates the relationship between SB and SEP. |
| Hypothesis 5b | (H5b) | Collaborating CMS positively and significantly mediates the relationship between SB and SEP. |
| Hypothesis 5c | (H5c) | Compromising CMS positively and significantly mediates the relationship between SB and SEP. |
| Hypothesis 5d | (H5d) | Avoiding CMS positively and significantly mediates the relationship between SB and SEP. |
| Hypothesis 5e | (H5e) | Accommodating CMS positively and significantly mediates the relationship between SB and SEP. |
