# Supplementary material for: Impact of supervisory behavior on sustainable employee performance: Mediation of conflict management strategies using PLS-SEM
Source: PLoS One. 2020 Sep 2;15(9):e0236650. doi: 10.1371/journal.pone.0236650 (PMC7467322; doi:10.1371/journal.pone.0236650)
Supplement: Attachment — Submitted filename: Review paper SEM and employee.docx [file pone.0236650.s004.docx]

**Editor’s comments to the paper:**

**Impact of Supervisory Behavior on Sustainable Employee Performance: Mediation of Conflict Management Strategies**

The paper investigates the relationship between supervisory behavior, conflict management strategies, and sustainable employee performance. Additionally, the study has examined the mediating effects of conflict management strategies between supervisory behavior and sustainable employee performance relationships. The significance of the model was assessed using the PLS-SEM. The paper adds in the current literature of being supervisory behavior as a critical predictor of sustainable employee performance. Additionally, the study adds in the current literature of PLS-SEM an assessment model for direct and mediation relationships. The study seems interesting and useful in the corresponding research field.

The paper, in general, in rough terms satisfies major rigor requirements that are demanded from Plos One. The red path remains relatively consistent throughout the paper; the main contributions and findings are clearly enough emphasized, analyzed, and justified. Moreover, in general, the paper is in most places, more or less sufficiently organized and written. So, to summarize, the contribution of the submitted paper seems relevant and useful for the researchers from the field.

However, the editor has detected some major issues that **are recommended** to be corrected prior to giving the paper to the reviewers and carrying out a further publishing process. Some major comments are:

1. The editorial board has expressed a certain doubt whether the paper meets PLOS ONE criteria for papers considering whether the limitations of observational studies have been acknowledged, including in the abstract; whether there are unsupported statements of causation; and whether the analysis is affected by confounding variables, a lack of generalizability, selective reporting, post hoc analyses, or data dredging. The editor thus appeals to the authors to check and reconsider this issue.
2. In the title, the term PLS-SEM is recommended to be included.
3. Please use the “structural equation” instead of “structured equation”.
4. The aesthetic look and resolution of figure 2 should be improved. The title of this figure is inadequate. Please use “The derived PLS-SEM model” or something similar. Denote the statistical significance of the parameters in Figure 2.
5. Also, it should be explained more clearly why the PLS-SEM modeling was used instead of the Covariance-based SEM (in most cases, the main reason should be a prediction – please look at *Hair, Chong (2016): An updated and expanded assessment of PLS-SEM in information systems research*).
6. In the literature review, the brief explanation of structural equation modeling and PLS-SEM methodology is missing, i.e., several sentences should be devoted to the methodologies used in the field.
7. Although it is relatively clear enough emphasized what the main contribution of the paper is, i.e., what has been done new, it is maybe not clear enough highlighted what are the main differences, if the novelties in this paper are compared with the newest state-of-the-art. Here, perhaps more precise borderline should be more clearly highlighted, such as for example, that this kind of research is a first attempt of “…”, which has not been designed or detected in the field yet. So, to summarize, please, clearly point to the research gap (in the introduction or literature review), that has been targeted by this paper, and mention where, how, and to what extent similar studies have been conducted, with the precise border between this study, and the other studies.
8. When introducing the hypotheses, it might be perhaps convenient to put them into some transparent, nice-looking table at the end of their explanation.
9. Before the appearance of Figure 1, please immediately and clearly define symbols of all variables (i.e., the original indicator variables and the latent factors) with adequate mathematical rigor. To do this, I recommend the Mathtype (please use it for all cases when presenting or mentioning any of the variables throughout the paper). In figure 1, please include all clusters of indicators in the sense that the nature of variables and causal paths among constructs and items are immediately evident (formative, reflective?).
10. In the methodological section, I miss a short description of structural equation modeling in general sense, as well as the PLS-SEM methodology in a specific sense.
11. When explaining the details of the survey, please include a brief, compact table of its properties and characteristics.
12. I am missing some of the basic descriptive statistics for the items, i.e., the mean, std, skewness, kurtosis,.., which would more clearly show the character and the level of non-normality of the data.
13. Please insert line numbers throughout all the paper.
14. Regarding all issues about statistical modeling and reporting of statistical results, the authors are invited **to synchronize** their work with the guidelines and rules of thumb of the afore-mentioned paper:
1. *Hair, Chong (2016): An updated and expanded assessment of PLS-SEM in information systems research*.

Moreover, it would be appropriate to look at the following papers as well:

2. *Jörg Henseler & Christian M. Ringle & Marko Sarstedt (2015): A new criterion for assessing discriminant validity in variance-based structural equation modeling;*
3. *Dijkstra, Henseler (2015): Consistent and asymptotically normal PLS estimators for*

*linear structural equations, and*

4. *Dijkstra, Henseler (2015): Consistent Partial Least Squares Path Modeling.*

At this very moment, there are several issues detected in the present paper, that are not synchronized with the newest findings of the just-mentioned papers of leading World’s experts regarding the SEM modeling. For instance, just to mention some issues:

**AD1.** According to the paper: *A new criterion for assessing discriminant validity in variance-based structural equation modeling*, authors Jörg Henseler & Christian M. Ringle & Marko Sarstedt (2015), the “…*the heterotrait-monotrait ratio (HTMT) of the correlations, which is the average of the heterotrait-heteromethod correlations (i.e., the correlations of indicators across constructs measuring different phenomena), relative to the average of the monotrait-heteromethod correlations (i.e., the correlations of indicators within the same construct)….*” should be concerned when a discriminant validity is tried to be accessed. The authors of this paper also emphasize: *“…Fornell-Larcker criterion and the assessment of the cross-loadings fail to reliably uncover discriminant validity problems in variance-based SEM…”*. Please, take into account this issue, i.e., include the HTMT besides the Fornell-Larcker criterion and the estimated cross-loadings.

**AD2.** In the *Hair, Chong (2016)* paper, there is a table I. regarding the differences between PLS-SEM and CB-SEM about rules of thumb for choosing the SEM method:

Thus, the major rules of thumb for choosing the SEM method are:

1. *The research objective is exploratory or confirmation of theory based on total variance.*

*2. The objective of the analysis is a prediction.*

*3. The measurement philosophy is estimation with the composite factor model using a total variance.*

*4. The research objective is to explain the relationships between exogenous and endogenous constructs.*

*5. The structural and/or measurement models are complex (many constructs and many indicators).*

*6. Formatively measured constructs are specified in the research.*

*7. The preferred method when the sample size is small (about 100 samples). But PLS is also an excellent method for larger samples.*

*8. The data are not normally distributed.*

*9. The scaling of responses is ordinal or nominal.*

*10. The data is secondary/archival, particularly single-item measures.*

*11. The research objective is to use latent variable scores in subsequent analyses.*

*12. The structural model will be estimated with a higher-order construct that has only two first-order constructs.*

*13. The analysis involves a continuous moderator.*

*14. The investigation will examine the model for unobserved heterogeneity*

Be cautious that all points emphasized above are consistent with the research and reporting in this paper.

**AD3.** In the *Hair, Chong (2016)* paper, there is a sub-section *Critical issues in PLS-SEM IS applications* regarding the eight critical issues that must be conducted in the research and appropriately reported:

- *reasons are given for using PLS-SEM,*
- *model descriptive statistics,*
- *sampling characteristics,*
- *technical reporting,*
- *formative measurement metrics,*
- *reflective measurement metrics,*
- *structural model metrics, and*
- *additional analyses such as mediation, moderation, multi-group analyses, and common methods variance.*

**AD4.** In the Hair, Chong (2016) paper, the following paragraph is particularly important and should be perhaps briefly mentioned in the paper:

*The latent variable scores for CB-SEM are indeterminant – i.e., an infinite number of different sets of latent variable scores that will fit the model equally well are possible for a CB-SEM solution, which makes* ***CB-SEM unsuitable for prediction*** *(Hair et al., 2016, 2018).* ***In contrast, the PLS-SEM method always produces a single determinant score for each SEM composite for each observation****. Moreover, in CB-SEM prediction, the R2 is related to the proportion of common variance explained, whereas in PLS-SEM, the R2 is related to the proportion of total variance explained (Hair et al., 2018).* ***Thus, PLS-SEM is always the preferred SEM method when the research objective is prediction*** *and it is believed that this reason for selecting PLS-SEM rather than CB-SEM will increase considerably in the future.*

**AD5**: In the Hair, Chong (2016) paper, there is also Table X explaining **the best practices about reporting the PLS-SEM results**. This table should be at the top of the list of concerns that the authors should be cautious with the most significant possible attention. The snapshot of this table is as following:

The authors are invited that (at least) in rough terms take into consideration these issues from Table X, particularly regarding reporting the results of the final model (Besides R2 and t values of the estimated coefficients, Predictive relevance (Q2), and p values of the estimated coefficients’ significance), maybe also some other measures from Table X and/or those measures that are offered from the Smart-PLS documentation (<https://www.smartpls.com/documentation/algorithms-and-techniques/model-fit>) might have been perhaps included in the paper. Accordingly, the authors are invited to reconsider this issue, whether it is also appropriate to include some other, additional measures.

15. The used software must always be reported (Smart-PLS?).
