# Supplementary material for: Impact of supervisory behavior on sustainable employee performance: Mediation of conflict management strategies using PLS-SEM
Source: PLoS One. 2020 Sep 2;15(9):e0236650. doi: 10.1371/journal.pone.0236650 (PMC7467322; doi:10.1371/journal.pone.0236650)
Supplement: Attachment — Submitted filename: PLOS-Editors Suggestions.docx [file pone.0236650.s005.docx]

**Editor’s comments to the paper:**

**Impact of Supervisory Behavior on Sustainable Employee Performance: Mediation of Conflict Management Strategies**

The paper investigates the relationship between supervisory behavior, conflict management strategies, and sustainable employee performance. Additionally, the study has examined the mediating effects of conflict management strategies between supervisory behavior and sustainable employee performance relationships. The significance of the model was assessed using the PLS-SEM. The paper adds in the current literature of being supervisory behavior as a critical predictor of sustainable employee performance. Additionally, the study adds in the current literature of PLS-SEM an assessment model for direct and mediation relationships. The study seems interesting and useful in the corresponding research field.

The paper, in general, in rough terms satisfies major rigor requirements that are demanded from Plos One. The red path remains relatively consistent throughout the paper; the main contributions and findings are clearly enough emphasized, analyzed, and justified. Moreover, in general, the paper is in most places, more or less sufficiently organized and written. So, to summarize, the contribution of the submitted paper seems relevant and useful for the researchers from the field.

However, the editor has detected some major issues that **are recommended** to be corrected prior to giving the paper to the reviewers and carrying out a further publishing process. Some major comments are:

1. The editorial board has expressed a certain doubt whether the paper meets PLOS ONE criteria for papers considering whether the limitations of observational studies have been acknowledged, including in the abstract; whether there are unsupported statements of causation; and whether the analysis is affected by confounding variables, a lack of generalizability, selective reporting, post hoc analyses, or data dredging. The editor thus appeals to the authors to check and reconsider this issue.

We have included limitations of observational studies in limitations section and in the abstract as well.

Limitations:

There were a few limitations associated with the present study. The time constraint was there to collect more responses. Also, the financial aspects forced to collect responses only from the SMEs situated in twin cities Rawalpindi and Islamabad; hence, it is difficult to offer generalizability in this study. Consequently, future research may focus on collecting data from SMEs in other cities of Pakistan to offer better generalization of findings. The study mainly collected data from manufacturing SMEs randomly; the future studies can target other industries as well. Moreover, this study focused on cross-sectional data, we suggest to develop more in-depth knowledge through longitudinal studies, and to investigate relationships between the constructs. Also, to find the differences in the results of cross-sectional and longitudinal studies. This study collected data from 122 employees of SMEs; future studies can increase the sample size to get more diverse responses. Similarly, this study collected data from employees of SMEs; further studies can explore multiple relationships of the model between different hierarchal levels such as executives, middle-level managers, and workers. Different cultural contexts and different approaches to treating the data could diversify future studies. (Lines 526-39)

Abstract:

This study is based on cross-sectional data, more longitudinal studies can explore relationships between the constructs. (Line 43-45)

In this study we have tried to discuss any relationships, specifically to the constructs supported by the past studies in hypothesis development section.

We have stated the generalizability issue in the limitations section of the paper as follows:

Also, the financial aspects forced to collect responses only from the SMEs situated in twin cities Rawalpindi and Islamabad; hence, it is difficult to offer generalizability in this study. Consequently, future research may focus on collecting data from SMEs in other cities of Pakistan to offer better generalization of findings. (Lines 527-30)

We have tried in this study to avoid selective reporting by searching multiple databases to find relevant studies and analyze them in relations to our study. We have tried to discuss the proposed hypothesis in relation to past studies to provide an initial bases of prediction, followed by the data analysis to generate results. Multiple tests in SmartsPLS were conducted to evaluate the reliability and validity of data, and model fit were explained.

2. In the title, the term PLS-SEM is recommended to be included.

The revised title as follows:

Impact of Supervisory Behavior on Sustainable Employee Performance: Mediation of Conflict Management Strategies using PLS-SEM (Lines 1-3).

3. Please use the “structural equation” instead of “structured equation.”

We have revised “structured equation” to “structural equation” throughout the paper.

4. The aesthetic look and resolution of figure 2 should be improved. The title of this figure is inadequate. Please use “The derived PLS-SEM model” or something similar. Denote the statistical significance of the parameters in Figure 2.

We have revised the Figure 2’s quality and title as follows:

Figure 2: The derived PLS-SEM model

5. Also, it should be explained more clearly why the PLS-SEM modeling was used instead of the Covariance-based SEM (in most cases, the main reason should be a prediction – please look at *Hair, Chong (2016): An updated and expanded assessment of PLS-SEM in information systems research*).

We have discussed it in the literature review section of the paper as follows:

This study uses SEM as a standard reporting method to allow replicability and establish rigor. Partial least square structural equation model (PLS-SEM) has been used in different disciplines such as strategic management and marketing (Hair, Sarstedt, Ringle and Mena, 2012), operation and international management (Peng and Lai, 2012; Richter et al, 2016), accounting (Lee et al., 2011), tourism (do-Valle and Assaker, 2015), family business (Sarstedt et al., 2014), organization and group research (Sosik et al., 2009). Moreover, this study used PLS-SEM instead of covariance-based SEM to predict the dependent affected by latent variables. Furthermore, an increasing trend was seen in published articles using PLS-SEM (Hair and Chong, 2017). The rationale for the choice of PLS-SEM was as follows. First, in CB-SEM the scores of latent variables are indeterminant, which makes it unsuitable to use in predictive studies. In comparison, PLS-SEM produces “a single determinant score for each SEM composite for each observation” (Hair et al., 2018). Secondly, in CB-SEM R2 relates to the proportion of common variance explained. In contrast in PLS-SEM R2 relates to the total variance explained (Hair et al., 2018). (Lines 175-83).

6. In the literature review, the brief explanation of structural equation modeling and PLS-SEM methodology is missing, i.e., several sentences should be devoted to the methodologies used in the field.

We have discussed the PLS-SEM methodology in literature review and methodology section. (Lines 175-88 and 329-35).

7. Although it is relatively clear enough emphasized what the main contribution of the paper is, i.e., what has been done new, it is maybe not clear enough highlighted what are the main differences, if the novelties in this paper are compared with the newest state-of-the-art. Here, perhaps more precise borderline should be more clearly highlighted, such as for example, that this kind of research is a first attempt of “…”, which has not been designed or detected in the field yet. So, to summarize, please, clearly point to the research gap (in the introduction or literature review), that has been targeted by this paper, and mention where, how, and to what extent similar studies have been conducted, with the precise border between this study, and the other studies.

We have revised the contribution of the study as follows:

This study contributes as follows. We used a more inclusive discernment to explore the multifaceted mediation role of CMS on SEP. Past studies on CMS mostly examined its direct effects on constructs such as efficacy and performance (Alper et al., 2000), innovation performance (Song and Lim, 2006), interpersonal rewards and performance (Weider-Hatfield and Hatfield, 1996), job satisfaction, cognition of action taking and sleep disorder (Way, Jimmieson, and Bordia, 2014). Moreover, previous studies have found positive relationship of SB and CMS ((Kodikal et al., 2014; Thomas, 1992; Zhao, Thatcher, and Jehn, 2019; Sabanci, Sahin, and Özdemir, 2016), employee performance and SEP (Kohli, 1989; Gilbreath and Benson, 2004; Fatima and Azam, 2016; Jiang et al., 2017; Ellinger, Ellinger, and Keller, 2003). However, this study uniquely examines the mediation role of CMS between the relationship of SB and SEP. Finally, this study enriches the literature on SB, CMS and SEP and provides substantial practical implications for managers at SMEs to enhance SEP. (Lines 86-97)

8. When introducing the hypotheses, it might be perhaps convenient to put them into some transparent, nice-looking table at the end of their explanation.

we have added a table exhibiting all hypothesis. (Annexure 1, lines 763-64)

9. Before the appearance of Figure 1, please immediately and clearly define symbols of all variables (i.e., the original indicator variables and the latent factors) with adequate mathematical rigor. To do this, I recommend the Mathtype (please use it for all cases when presenting or mentioning any of the variables throughout the paper). In figure 1, please include all clusters of indicators in the sense that the nature of variables and causal paths among constructs and items are immediately evident (formative, reflective?).

Suggested changes were made throughout the manuscript including Figure 1.

10. In the methodological section, I miss a short description of structural equation modeling in general sense, as well as the PLS-SEM methodology in a specific sense.

This study used partial least square (PLS) modeling to analyze the conceptual model. We used PLS path modeling because it has received waste application in management and related fields (Hairet al., 2012; Kura, 2016; Kura et al., 2015). This study aimed to predict the dependant variable, hence PLS path modeling was considere a suitable investigative method (Hair et al., 2011). Scholars suggests PLS as “most fully developed and general system” (McDonald, 1996) concerning the “variance based structured equation modeling” method (Ringle et al., 2015). Therefore, the data were further analyzed using Smart-PLS 3 to examine the proposed relationships. (Lines 329-35).

11. When explaining the details of the survey, please include a brief, compact table of its properties and characteristics.

We have added a table explaining details of survey (demographic information of repondents). (Lines 326-28).

12. I am missing some of the basic descriptive statistics for the items, i.e., the mean, std, skewness, kurtosis,.., which would more clearly show the character and the level of non-normality of the data.

We have added the basic descriptive statistics for the items and variables. (Lines 421-37).

13. Please insert line numbers throughout all the paper.

We have added line numbers throughout all the paper.

14. Regarding all issues about statistical modeling and reporting of statistical results, the authors are invited **to synchronize** their work with the guidelines and rules of thumb of the afore-mentioned paper:
1. *Hair, Chong (2016): An updated and expanded assessment of PLS-SEM in information systems research*.

Moreover, it would be appropriate to look at the following papers as well:

2. *Jörg Henseler & Christian M. Ringle & Marko Sarstedt (2015): A new criterion for assessing discriminant validity in variance-based structural equation modeling;*
3. *Dijkstra, Henseler (2015): Consistent and asymptotically normal PLS estimators for*

*linear structural equations, and*

4. *Dijkstra, Henseler (2015): Consistent Partial Least Squares Path Modeling.*

At this very moment, there are several issues detected in the present paper, that are not synchronized with the newest findings of the just-mentioned papers of leading World’s experts regarding the SEM modeling. For instance, just to mention some issues:

**AD1.** According to the paper: *A new criterion for assessing discriminant validity in variance-based structural equation modeling*, authors Jörg Henseler & Christian M. Ringle & Marko Sarstedt (2015), the “…*the heterotrait-monotrait ratio (HTMT) of the correlations, which is the average of the heterotrait-heteromethod correlations (i.e., the correlations of indicators across constructs measuring different phenomena), relative to the average of the monotrait-heteromethod correlations (i.e., the correlations of indicators within the same construct)….*” should be concerned when a discriminant validity is tried to be accessed. The authors of this paper also emphasize: *“…Fornell-Larcker criterion and the assessment of the cross-loadings fail to reliably uncover discriminant validity problems in variance-based SEM…”*. Please, take into account this issue, i.e., include the HTMT besides the Fornell-Larcker criterion and the estimated cross-loadings.

We have inculded the HTMT ration in the paper and consulted the suggested papers to meet the rule of thumb.

Scholars Gold et al. (2001), Teo et al. (2008), Hair and Chong (2017) suggests to measure the multicollinearity in data by HTMT ratio, which should not be higher than 0.9. This study met the threshold exhibited in Table 3. (Lines 383-85 and 388-89).

Furthermore, we have carefully read the suggested paper and synchronized all of the results of the paper with suggested studies.

**AD2.** In the *Hair, Chong (2016)* paper, there is a table I. regarding the differences between PLS-SEM and CB-SEM about rules of thumb for choosing the SEM method:

Thus, the major rules of thumb for choosing the SEM method are:

1. *The research objective is exploratory or confirmation of theory based on total variance.*

*2. The objective of the analysis is a prediction.*

*3. The measurement philosophy is estimation with the composite factor model using a total variance.*

*4. The research objective is to explain the relationships between exogenous and endogenous constructs.*

*5. The structural and/or measurement models are complex (many constructs and many indicators).*

*6. Formatively measured constructs are specified in the research.*

*7. The preferred method when the sample size is small (about 100 samples). But PLS is also an excellent method for larger samples.*

*8. The data are not normally distributed.*

*9. The scaling of responses is ordinal or nominal.*

*10. The data is secondary/archival, particularly single-item measures.*

*11. The research objective is to use latent variable scores in subsequent analyses.*

*12. The structural model will be estimated with a higher-order construct that has only two first-order constructs.*

*13. The analysis involves a continuous moderator.*

*14. The investigation will examine the model for unobserved heterogeneity*

Be cautious that all points emphasized above are consistent with the research and reporting in this paper.

We have craefully revised and made sure that the choice of PLS-SEM meets the rule of thumb in our study.

**AD3.** In the *Hair, Chong (2016)* paper, there is a sub-section *Critical issues in PLS-SEM IS applications* regarding the eight critical issues that must be conducted in the research and appropriately reported:

- *reasons are given for using PLS-SEM,*
- *model descriptive statistics,*
- *sampling characteristics,*
- *technical reporting,*
- *formative measurement metrics,*
- *reflective measurement metrics,*
- *structural model metrics, and*
- *additional analyses such as mediation, moderation, multi-group analyses, and common methods variance.*

We have carefully mentioned above points in the manuscript.

**AD4.** In the Hair, Chong (2016) paper, the following paragraph is particularly important and should be perhaps briefly mentioned in the paper:

*The latent variable scores for CB-SEM are indeterminant – i.e., an infinite number of different sets of latent variable scores that will fit the model equally well are possible for a CB-SEM solution, which makes* ***CB-SEM unsuitable for prediction*** *(Hair et al., 2016, 2018).* ***In contrast, the PLS-SEM method always produces a single determinant score for each SEM composite for each observation****. Moreover, in CB-SEM prediction, the R2 is related to the proportion of common variance explained, whereas in PLS-SEM, the R2 is related to the proportion of total variance explained (Hair et al., 2018).* ***Thus, PLS-SEM is always the preferred SEM method when the research objective is prediction*** *and it is believed that this reason for selecting PLS-SEM rather than CB-SEM will increase considerably in the future.*

We have discussed the suggested paragraph in the manuscript as follows:

The rationale for the choice of PLS-SEM was as follows. First, in CB-SEM the scores of latent variables are indeterminant, which makes it unsuitable to use in predictive studies. In comparison, PLS-SEM produces “a single determinant score for each SEM composite for each observation” (Hair et al., 2018). Secondly, in CB-SEM R2 relates to the proportion of common variance explained. In contrast in PLS-SEM R2 relates to the total variance explained (Hair et al., 2018). (Lines 183-88).

**AD5**: In the Hair, Chong (2016) paper, there is also Table X explaining **the best practices about reporting the PLS-SEM results**. This table should be at the top of the list of concerns that the authors should be cautious with the most significant possible attention. The snapshot of this table is as following:

The authors are invited that (at least) in rough terms take into consideration these issues from Table X, particularly regarding reporting the results of the final model (Besides R2 and t values of the estimated coefficients, Predictive relevance (Q2), and p values of the estimated coefficients’ significance), maybe also some other measures from Table X and/or those measures that are offered from the Smart-PLS documentation (<https://www.smartpls.com/documentation/algorithms-and-techniques/model-fit>) might have been perhaps included in the paper. Accordingly, the authors are invited to reconsider this issue, whether it is also appropriate to include some other, additional measures.

All of the suggested tests in the table were duly reported in the manuscript.

15. The used software must always be reported (Smart-PLS?).

We have reported the software used as follows:

The data were further analyzed using Smart-PLS 3 to examine the proposed relationships. (Lines 334-35).
