# Supplementary material for: Impact of supervisory behavior on sustainable employee performance: Mediation of conflict management strategies using PLS-SEM
Source: PLoS One. 2020 Sep 2;15(9):e0236650. doi: 10.1371/journal.pone.0236650 (PMC7467322; doi:10.1371/journal.pone.0236650)
Supplement: Attachment — Submitted filename: REPORT1_REVIEW.docx [file pone.0236650.s006.docx]

REVIEW REPORT

Strengths

The enthusiasm of the authors towards the topic is seen throughout the manuscript. The Manuscript is also detailed and contributes immensely to literature on managing conflicts among employees in SMEs. The authors succeeded in identifying the relevance of their research. However, I found the writing and organization of the manuscript uneven. There are problems in all the sections, including your mediation analysis.

Weaknesses

ABSTRACT

1. The Abstract will benefit from English Language editing. You mixed up the tenses in the Abstract. For instance, in lines 32 to 34, the purpose…is to investigate…Additionally, the study has examined…Please improve the language.
2. In line 38 “relationship”, needs correction. Lines 43 and 44 need correction. The study…more longitudinal…constructs. Line 45 needs correction. It should read as “…the study adds to the current…

INTRODUCTION

1. The Introduction will benefit from English Language editing. For instance, check the preposition after “add”. Check throughout for this problem.
2. In line 71, change the word “primitively” to a more suitable synonym.
3. In line 87, is the mediation role of CMS on SEP, or on the relationship between SB and SEP?
4. Restructure sentence in line 86.
5. Authors should provide brief information on PLS-SEM in the introduction.

LITERATURE REVIEW

1. The study has more than one hypothesis, so correct the spelling in line 98. Check throughout for this problem.
2. In lines 154 and 155 … brake down, and dysfunctional… need correction.
3. Line 159, you CANNOT start a sentence with a citation: (De-Dreu et al., 1999). Write the name of the author(s), then add the citation. Check throughout for this problem.
4. Adding the review on the PLS-SEM to the review on the CMS is not right. PLS-SEM and CMS are different. Authors should have a separate sub-heading for PLS-SEM.
5. This: **“In the literature review, the brief explanation of structural equation modeling and PLS-SEM methodology is missing, i.e., several sentences should be devoted to the methodologies used in the field”** **should be included as indicated earlier**. Authors only showed the reason for using PLS-SEM. You should provide a review of structural equation modeling as well as PLS-SEM methodology.

HYPOTHESES DEVELOPMENT

1. Line 194…concerning to… need correction. The sentence in line 196 needs correction. The sentence in lines 203 to 205 needs correction. Line 209, change “ample” to a more appropriate synonym. Line 213 needs correction. I suggest “Backed by evidence from Mehboob and colleagues (Mehboob et al., 2011), this study… Line 229 ‘unsolved’ needs correction. Line 235, I suggest authors add ‘Therefore’ after the citation before introducing the statement for the hypothesis. Line 253…other’s… needs correction. Line 256, if CMS is considered as one entity, check ‘affect’ and correct the subject-verb agreement as needed. Authors should correct the sentence in line 259. Introduce linking words between ‘issues’ and ‘meant’. For instance …issues; yet these conflicts are meant… Restructure sentences in lines 261 to 263. Studies…performance. Lines 263 and 266…‘in’… needs correction. Line 277… performance; this …Authors should change this punctuation. Line 285 needs correction. Check and insert the appropriate verb.
2. Literature on the hypotheses (H5a to H5e) for the various mediating variables is not convincing. Your literature refers to counterproductive work behavior. There is no correlation between the literature provided and how the various variables can mediate the relationship between SB and SEP. I missing the point whereby counterproductive can be synonymous with either SB or SEP. The authors need to justify how the dimensions of CMS can mediate the link between SB and SEP. Again, supposedly, H5A to H5e are different from H4. If possible, authors should provide brief literature before they begin to explain these dimensions.

RESEARCH METHODOLOGY

1. Ethical Statement: I suppose your research is only one study, so why do authors use ‘studies’. The whole statement needs to be corrected.
2. Separate the Ethical statement from the methods. Combining them and providing a sub-heading as ‘Ethical Statement’ is confusing.
3. Lines 309 to 314, what are the contributions of these to the research methodology. These sentences should be part of the introduction.
4. The Research Methodology should start from lines 315. Insert the ‘Ethical Statement’ before ‘4. Analysis and Results’.
5. Line 312… (25 percent); overall…Change punctuation. I suggest “(25 percent). Overall… Line 313 is not clear. It needs to be corrected. Line 315 … targets… needs correction. Line 316…SMEs 65.4 percent can be changed to ‘SMEs at 65.4 percent. Line 321 change ‘usable’ to a more appropriate synonym. Lines 322 and 333, The study… concerns… performance, needs correction. Line 330, change …concerning… to make the sentence meaningful.
6. Line 326 to332 should be put under separate sub-heading. For instance, ‘Data Analysis’
7. I missed important details about the data collection procedure in your methodology. Were there ethical considerations regarding your respondents for this study?
8. It is appropriate to insert Table 1 before the Data Analysis. Check the results on the demographic information. The percentage of ‘industry’ is more than 100%.
9. Authors provided a Cronbach alpha value (0.904) under the measure for SEP. However, another Cronbach alpha (0.931) is provided for SEP in TABLE 2. Authors should clarify this. Besides, authors should be consistent in their representation of the results. If they choose to add Cronbach alpha to the measures, as depicted for SEP, then it should be the same for all measures.

ANALYSIS AND RESULTS

1. I suggest authors change the heading to ‘Results’ since there is a suggested sub-heading: ‘Data Analysis’.
2. Line 352…pas… needs correction. Line 380 and 381, insert appropriate punctuation after ‘Scholars’ and revise the subject-verb agreement (Scholars… suggests…) in the sentence. Line 393 needs to be corrected. Line 402, the use of if’ is incorrect. Revise sentence in line 444…show that; H1 shows that…
3. According to Hair’s work, indicator loadings should be equal to or more than 0.70. However, in your paper, you stated a threshold of 0.40 to 0.70, meaning values more than 0.70 is unacceptable. Please refer to the information provided earlier for your major revision, and provide the precise statement.
4. Please provide the full meanings for the abbreviations in Table 2. Do the same for other Tables.
5. Table 3 provides results on the Discriminant validity. Yet Table 5 is entitled Discriminant validity….. It is a bit confusing. Table 5 is on correlations among the constructs, which is different from the correlation to check the discriminant validity. Authors should clarify this. Authors’ denotations of the significant levels are incorrect (eg. ** p-value <0.05).

RESULTS

1. Your direct analysis may be correct. However, authors’ mediation analysis is not acceptable. Please refer to the following works on mediation analysis:

- *Preacher, K. J., & Hayes, A. F. (2008). Asymptotic and resampling strategies for assessing and comparing indirect effects in multiple mediator models. Behavior research methods, 40(3), 879-891*.
- *Zhao, X., Lynch Jr, J. G., & Chen, Q. (2010). Reconsidering Baron and Kenny: Myths and truths about mediation analysis. Journal of consumer research, 37(2), 197-206.*
- *Shrout, P. E., & Bolger, N. (2002). Mediation in experimental and nonexperimental studies: new procedures and recommendations. Psychological methods, 7(4), 422.*
- *Nitzl, C., Roldan, J. L., & Cepeda, G. (2016). Mediation analysis in partial least squares path modeling. Industrial management & data systems.*

Authors can also refer to David Mackinnon’s works for mediation analysis.

2. Authors provided results on kurtosis and skewness in Table 6. The skewness and kurtosis with their standard errors should be (+ or – 1.96) which indicates normality in the data. But some of the values are more than the threshold. Authors should check and make necessary corrections.

DISCUSSION AND CONCLUSION

1. Line 466…‘supports’… needs correction. Line 467…‘shows’… needs correction. Line 471 ‘effects’… needs correction. Line 489… ‘the five’… needs correction. Line 490…‘individual’… needs correction. The sentence in line 496 needs to be revised. Line 499 …‘affecting’… needs to be corrected. Line 500, Finally, all five…situations, needs to be corrected.
2. Lines 477 to 479 reveal that adaptive performance was affected by SB and SEP. Yet, authors used adaptive performance as a dimension of SEP. I missed the point where authors measured the effect of SEP on adaptive performance. Please clarify this and revise it as needed.

LIMITATIONS AND FUTURE RESEARCH DIRECTIONS

1. In line 522 ‘The time constraint was there to collect more responses’. This is confusing. How were authors able to collect more responses if they were bounded by time?
2. Lines 523 ‘financial aspects forced’… is not clear. Please authors should clarify.

GENERAL COMMENTS

The English language is fairly good. However, the manuscript needs a high level of English language editing. Please go through the whole manuscript to refine the language and make all corrections where necessary. Authors may need a native English speaker to do this properly. There is an article here. However, it demands a major revision.
